# Radiation Dose to Newborns in Neonatal Intensive Care Units

**DOI:** 10.5812/iranjradiol.8065

**Published:** 2012-09-17

**Authors:** Mohammad Taghi Bahreyni Toossi, Malakeh Malekzadeh

**Affiliations:** 1 Medical Physics Research Center, Faculty of Medicine, Mashhad University of Medical Sciences, Mashhad, Iran; 2 Nursing and Allied Health Faculty, Semnan University of Medical Sciences, Semnan, Iran

**Keywords:** Intensive Care Units, Neonatal, Radiation Dosimetry

## Abstract

**Background:**

With the increase of X-ray use for medical diagnostic purposes, knowing the given doses is necessary in patients for comparison with reference levels. The concept of reference doses or diagnostic reference levels (DRLs) has been developed as a practical aid in the optimization of patient protection in diagnostic radiology.

**Objectives:**

To assess the radiation doses to neonates from diagnostic radiography (chest and abdomen). This study has been carried out in the neonatal intensive care unit of a province in Iran.

**Patients and Methods:**

Entrance surface dose (ESD) was measured directly with thermoluminescent dosimeters (TLDs). The population included 195 neonates admitted for a diagnostic radiography, in eight NICUs of different hospital types.

**Results:**

The mean ESD for chest and abdomen examinations were 76.3 µGy and 61.5 µGy, respectively. DRLs for neonate in NICUs of the province were 88 µGy for chest and 98 µGy for abdomen examinations that were slightly higher than other studies. Risk of death due to radiation cancer incidence of abdomens examination was equal to 1.88 × 10 ^-6^ for male and 4.43 × 10 ^-6^ for female. For chest X-ray, it was equal to 2.54 × 10 ^-6^ for male and 1.17 × 10 ^-5^ for female patients.

**Conclusion:**

DRLs for neonates in our province were slightly higher than values reported by other studies such as European national diagnostic reference levels and the NRPB reference dose. The main reason was related to using a high mAs and a low kVp applied in most departments and also a low focus film distance (FFD). Probably lack of collimation also affected some exams in the NICUs.

## 1. Background

Most of the performed diagnostic X-ray examinations during hospitalization in the neonatal intensive care units (NICU) comprise imaging of the respiratory and gastrointestinal systems; namely, the chest and abdomen examinations. Several literature reports emphasize that the risk of cancer from exposure is inversely proportional with age, meaning that the radiosensitivity of a newborn is assumed to be greater than a mature child or an adult (1, 2); therefore, the risk of radiation induced malignancy is increased (3). Preterm birth rate has risen by nearly 16% since 1990 (1). At present, about 11% of all newborns in North America and Africa are born premature (4). At the same time, the survival of neonates has increased significantly. Most of these neonates will require multiple X-rays during their neonatal course which depends on the underlying disease and these standardized images permit health care providers to interpret accurately and formulate appropriate interventions. Children are more susceptible to low levels of radiation because they possess many rapidly dividing cells, in which repair of mutations is less efficient than in resting cells. Since mutated DNA by radiation cannot be repaired while the cell continues to divide; therefore, the DNA remains damaged. Smaller children potentially may receive a greater effective dose. Because of their size, more body tissues may be irradiated than larger children or adults (5). The risk of cancer induction per unit of dose is believed to be 2-3 times higher than that of the average population and 6-9 times higher than the risk from an exposure of a 60-yearold (6). Diagnostic radiology examinations need to be optimized so that doses received by the patient are not higher than needed to obtain the required diagnostic information. The International Commission on Radiological Protection (ICRP) has encouraged “authorized bodies to set diagnostic reference levels that best meet their specific needs and are consistent for the regional, national or local area to which they apply.” DRLs were determined through the 3rd quartile of the distribution of mean ESD values (7). Therefore, DRL is not dose limit, but a guide for doing well. Until now, no DRL has been established for neonate radiologic examination in Iran. The extent of exposure depends particularly on the exposure parameters such as applied potential (tube voltage in kV), current-time product (mAs), focus to skin distance (FSD) and the radiographic techniques (determination of examination field size borders). Incorrect determination of these parameters affects the dose to organs as well as the image quality. Similar studies were performed by Smans et al. in Belgium (6) and Olgar et al. in Turkey for determining neonate’s dose by usage of TLD (8). On the other hand, there are some rare studies in pediatric dosimerty in Iran which is very new. In our previous survey, mean ESD was measured for neonates in five random departments in Mashhad (9). In addition radiation dosimetry for fifty neonates, in five hospitals was carried out by Faghihi et al. in Shiraz (10). The purpose of this study was to evaluate diagnostic reference levels at the neonatal intensive care unit (NICU).

## 2. Objectives

The present study was focused on chest and abdomimal radiographs, since these are the most common radiographic examinations performed at the NICUs.

## 3. Patients and Methods

This study was performed during a period of 6 months in eight NICUs located in different types of maternity, pediatric, general and educational hospitals. The studied population included 195 neonates of both genders with different neonatal illnesses. The mean weight of the neonates was 2650g. The radiographic parameters such as applied potential (kVp), current-time product, film to focus distance (FFD) and neonate data were recorded. In the NICU, radiographs are taken with mobile X-ray units. The quantity measured in this survey was Entrance Surface Dose (ESD) at the point of intersection of the beam axis with the patient surface (skin), including backscattered radiation. ESD can be measured by placing LiF;Mg, Ti thermoluminescent dosimeters (TLD-100) on the skin of the patient during X-ray examinations. The TLDs are near tissue equivalent and are therefore not visible on the image. TLD calibration was done by irradiation of a group of TLDs, by diagnostic X-rays (80 kV, total filtration of 3.0 mmAl), to a known dose (mGy range) measured by a 6 cm ion chamber and Radcal monitor (model 9015). Two TLD chips were used for each neonate and also for measuring the background radiation, TLD chips were later read by Harshaw 3500 TLD Reader. Incident air KERMA, K ^a.i^ was calculated from ESD values for estimating the risk with PCXMC: 

(1) K^a.i^ = ESD/BFS

Where BSF is the backscatter factor. In these calculations, a backscatter factor of 1.1 is used (6). Incident air KERMA, K ^a.i^ was used as the input factor to PCXMC software to calculate the risk of death due to radiation cancer incidence originated from chest and abdomen X-ray examinations. The PCXMC Monte Carlo program (PCXMC2.0, STUK, Radiation and Nuclear Safety Authority, Helsinki, Finland) uses hermaphrodite mathematical phantoms based on the models of Cristy (1980), which are adjustable for weight, height and ages of 0, 1, 5, 10, 15 years and adult. In order to assess the risk of radiation induced cancer death for a given patient, the user needs to enter correct patient data for the ‘Age’, ‘Gender’ and ‘Mortality Statistics’ (Euro-American, Asian or Finnish) of the patient. An alternative method is to use computational dosimetry techniques that simulate medical X-ray exposures on computerized phantoms and use Monte Carlo radiation transport codes to calculate the energy deposited in each organ (11). “Mobile radiography” equipment has wheels that enable it to be moved to a specific location while “portable radiography” is the unit that may be carried to a destination. In hospitals, portable radiography is verbally used instead of mobile radiography. The mobile X-ray machines in participating hospitals were Siemens in center 1, 4, 5 and 8; Dyamax in center 2; Shimadzu in centers 3 and 7 and Toshiba in center 6.

## 4. Results

**Figure 1:**
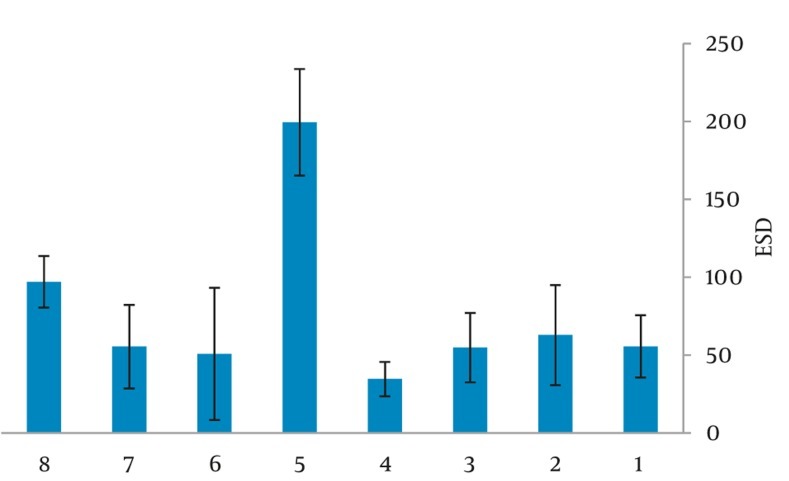
Mean ESD (μGy) for chest examination in 8 centers

**Figure 2:**
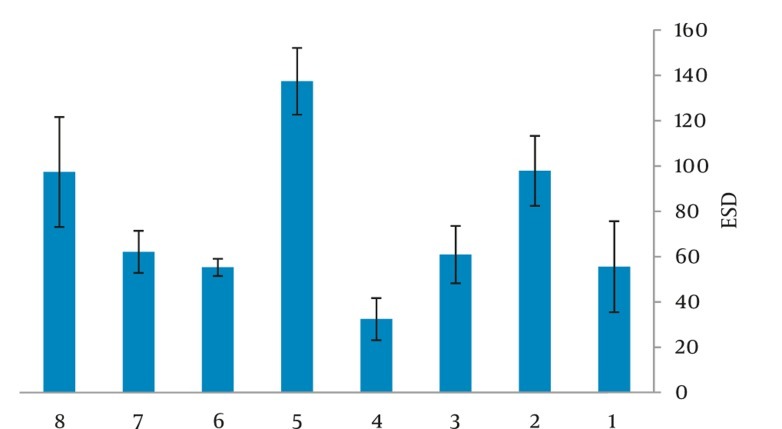
Mean ESD (μGy) for abdomen examination in 8 centers

Figure 1 and Figure 2 show the mean ESD for chest and abdomen examinations in NICUs produced by Microsoft Excel Software. Center no. 5 (a general hospital) shows the highest value, 199 µGy for chest exam while we observed the minimum ESD in center no. 4 (a maternity hospital) equal to 35 µGy. For Figure 2, the highest value of mean ESD was acquired in center no. 5 just 137 µGy minimum dose (ESD) was equal to 32 µGy obtained in a maternity hospital. For both examinations, the range of applied kvp values were 41-61 and 41-62 kVp, respectively and the current-time product values were 0.5-2.8 and 0.5-4 mAs. Table 1 illustrates the Risk of Exposure Induced Cancer Death (REID) for chest and abdomen examinations calculated by PCXMC software which uses the model developed by the BEIRVII committee (Committee on the Biological Effects of Ionizing Radiations, BEIR 2006). Table 1 illustrates REID of abdomen examination which was equal to 1.88 × 10^-6^ for boys and 4.43 × 10^-6^ for girls while for chest X-ray, it was equal to 2.54 × 10^-6^ and 1.17 × 10^-5^ for boys and girls, respectively. REID was higher for girls than the boys, which could mean that girls are more radiosensitive than boys.

**Table 1:** Risk of Exposure Induced Cancer Death of Examinations Based on Gender

	Risk of Exposure Induced Cancer Death (REID) for 0.060 mGy arising from chest examination
**Gender**	
Male	2.54× 10^-6^
Female	1.17× 10^-5^
	Risk of Exposure Induced Cancer Death (REID) for 0.048 mGy arising from abdomen examination
**Gender**	
Male	1.88 × 10^-6^
Female	4.43 × 10^-6^

## 5. Discussion

We observed minimum ESD in center no. 4 (a maternity hospital), due to the high kVp (61-62) and low mAs (0.5) used. In center no. 5 (a general hospital), a higher ESD was obtained, radiation field was 1000-1200 cm2, lack of proper collimation was probably the main reason for the higher patient dose. Short FFD (58cm) caused higher mean ESD in center no. 8 (a general hospital) for both types of examinations. In center no. 2 (an educational hospital), patients who underwent abdominal radiographies received relatively higher ESD; this may be associated with the selection of high mAs equal to 4. During this study, center no. 3 used CR (Computed Radiography) technologies while the other used conventional screen film (speed 400). Data given in Figure 1 and 2 were optimized for screen film systems and not for computed radiography systems used nowadays. The inappropriate field size is the most important mistake in the pediatric radiographic technique. A field which is too large will not only impairs the image contrast and resolution by increasing the amount of scattered radiation, but more importantly results in unnecessary irradiation of the body. However, correct beam limitation requires proper knowledge of the external anatomical landmarks by the technician. This illustrates the need for both theoretical and practical teaching of the technicians. Deviation between the radiographic parameters for each NICU indicates that examinations were taken by a large number of radiographers. It means that technicians who practice in NICUs had no special training for the job. although former studies recommended that every X-ray department should assign a group specializing in pediatric imaging. The differences found in radiographic parameters may cause variations in the doses to which neonates are exposed. It should be noted that the radiographic voltage may depend on the type and age of the X-ray tube. Table 2 provides patients' data, mean ESD and DRL comparison with former similar investigations. Faghihi et al. determined the values of ESD, dose area products and energy imparted by three methods including direct method [using thermoluminescence dosimetry (TLD)], indirect method (using tube output) and Monte Carlo (MC) method. Their results indicate that the mean ESD per radiograph estimated by direct, indirect and MC methods are 56.6 + 4.1, 50.1 + 3.1 and 54.5 m + 3.3 µGy, respectively. They have not illustrated that these doses were related to which exam, but they have indicated dose per radiograph (10). In our previous study, the mean ESD was determined for neonates under stationary and mobile units. The mean ESD was 191.85 µGy for the chest and 197.30 µGy for the abdomen. The main reason was usage of grid for the infant in the hospitals (9). In addition, Smans et al. categorized the newborn infants into three birth weight groups (< 1000 gr, 1000-2500 gr and > 2500gr) and found the mean ESD is increased with the weight at birth and reported the mean ESDs of 28, 33 and 52 µGy for the groups, respectively. So for comparison, we chose their third group based on the infants’ weight at birth (6). Olgar et al. also used TLD for measuring ESDs, their results showed that neonates received acceptable doses from common radiological examinations in Turkey (8). Brindhaban et al. reported the birth weight range was between 750 and 2000gr for infants (12). However, in this study, the mean ESD for chest and abdomen examination were 76.3 µGy and 61.5µGy, respectively; also DRLs for neonates in our province were 88 µGy for chest and 98µGy for abdomen examinations that were slightly higher than other studies; European national diagnostic reference levels of 80 µGy for mobile chest radiographs and the NRPB reference dose of 50 µGy for chest examination. The main reason was related to using a high mAs and a low kVp in most departments and also a low FFD. Probably lack of collimation affected some exams in NICUs. Increased kVp (reduced mAs) causes greater penetration and less absorption then reduced patient dose for a constant film density. We should mention that the range of applied potential values was 41-61 kVp for chest exam, while the CEC recommendations are (60-65 kVp for neonatal, 70-80 kVp for children up to 5 years and 100-120 kVp for older children) (13, 14). The recommended kVp causes less contrast, but better assessment of the lung parenchyma. Lower kVp is necessary if looking for bone details (15). It is necessary to encourage radiographers to use suitable exposure factors and good collimation. Variations in size are marked not only for adults, but also for pediatric patients and the use of a single reference size is impractical to determine exposures factors. The large variation in ESD values indicate that patient dose can be diminished by paying more attention to exposure factors (kV, mAs), without loss of image quality, pointing to the importance of quality control programs. Staff should try to nurture using shielding for neonates such as additional collimation to achieve non-regular field shapes by placing lead sheets on the top of the incubator. Therefore, for dose reduction, several actions at national as well as international levels should be taken. Those actions should prevent the increasing risk of long-term effects to neonates, a unique group which undergoes multiple diagnostic examinations. All in all, the examination technique in pediatric radiology should be optimized. Establishing local diagnostic reference levels (LDRL) in each department or maybe each X-ray room can be an effective way for paying attention to patient dose and optimization and it is much more effective if national reference dose levels (NDRL) can be established for pediatrics.

**Table 2:** Comparison Between DRL c s and Mean ESD c s Arising from Examinations with Previous Studies

**Mean ESD, μGy**											**DRL, μGy**	
Examination	Mean weight, gr	Mean height , cm	No. patient	Mean ESD This Work	DRL This Work, μGy	Faghihi et al., 2011 (10)	Malekzadeh et al., 2009(9)	Smans et al., 2008(6)	Olgar et al., 2008(8)	Brindhaban et al., 2004 (12)	CEC et al., 1996(13)	Hart et al.,2000(14)
Chest	2630	48.75	120	76.3	88	56.6 b	84.63	52	67	51-102	80	50
Abdomen	2666	49.72	75	61.5	98	NA	NA	NA	65	58-102	NA ^a^	NA ^a^

^a^Not available

^b^ESD per radiograph

^c^Abbreviations: DRL, diagnostic reference level; ESD, Entrance surface Dose

For decreasing dose, several actions should be pursued: remarking the ALARA (As Low as Reasonable Achievable) concept, defining national guidelines for good neonate radiography and, retraining radiographers to be specialized for neonatal imaging and neonatal ionization radiation hazards. The medical physicist is the best suited individual to monitor patient doses and to reduce them (if possible) without substantially compromising the efficacy of diagnostic procedures. Medical physicists are also in charge of patient safety including radiation, mechanical and electrical safety.
